# Association of lithium versus valproate initiation with all-cause mortality and incident dementia: a multinational real-world cohort study

**DOI:** 10.3389/fneur.2026.1847655

**Published:** 2026-09-22

**Authors:** Da-Han Hou, Yu-Hsien Wu, Ming-Ju Wu, Shiau-Shian Huang, Po-Hsun Hou

**Affiliations:** 1 Department of Medical Education, Taichung Veterans General Hospital, Taichung, Taiwan; 2 Department of Psychiatry, Taichung Veterans General Hospital, Taichung, Taiwan; 3 Division of Nephrology, Department of Internal Medicine, Taichung Veterans General Hospital, Taichung, Taiwan; 4 Department of Post-Baccalaureate Medicine, College of Medicine, National Chung Hsing University, Taichung, Taiwan; 5 Rong-Hsing Research Center for Translational Medicine, College of Life Sciences, National Chung Hsing University, Taichung, Taiwan; 6 School of Medicine, Chung Shan Medical University, Taichung, Taiwan; 7 Graduate Institute of Biomedical Sciences, College of Medicine, School of Medicine, China Medical University, Taichung, Taiwan; 8 College of Medicine, National Yang Ming Chiao Tung University, Taipei, Taiwan; 9 School of Public Health, National Defense Medical Center, Taipei, Taiwan; 10 Department of Psychiatry, Taipei Veterans General Hospital, Taipei, Taiwan; 11 Nankung Psychiatric Hospital, Keelung, Taiwan

**Keywords:** dementia, lithium, mortality, real-world evidence, valproate

## Abstract

**Background:**

Lithium and valproate are widely prescribed mood stabilizers, but their comparative long-term associations with mortality and dementia remain uncertain. We compared the risks of all-cause mortality and incident dementia among adults initiating lithium or valproate using a large multinational electronic health record network.

**Methods:**

We conducted a retrospective cohort study using the TriNetX Global Collaborative Network (2004–2024), a multinational electronic health record network. Adults aged ≥40 years initiating lithium or valproate were identified after excluding patients with pre-existing dementia, epilepsy, intracranial injury, kidney transplantation, advanced chronic kidney disease, or acute kidney injury. The primary analysis followed an intention-to-treat approach based on the first recorded prescription of lithium or valproate. Propensity score matching incorporated demographic, clinical, psychiatric, and treatment-related characteristics, resulting in 22,098 patients in each treatment group. A 30-day prescription-confirmed analysis and lag-time analyses were performed as supportive and sensitivity analyses, respectively.

**Results:**

During follow-up, 884 deaths occurred among lithium initiators and 1,585 among valproate initiators. The corresponding five-year Kaplan–Meier-estimated cumulative event probabilities for all-cause mortality were 6.61 and 10.65%, respectively. Lithium initiation was associated with a lower recorded risk of all-cause mortality than valproate initiation [hazard ratio (HR) 0.58, 95% confidence interval (CI) 0.53–0.63; *p* < 0.001]. Incident dementia occurred in 495 lithium initiators and 754 valproate initiators, with corresponding five-year Kaplan–Meier-estimated cumulative event probabilities of 3.51 and 4.85%, respectively. Lithium initiation was also associated with a lower recorded risk of incident dementia (HR 0.68, 95% CI 0.60–0.76; *p* < 0.001). Findings remained directionally consistent in the supportive prescription-confirmed and lag-time analyses.

**Conclusion:**

Lithium initiation was associated with lower recorded risks of all-cause mortality and incident dementia than valproate initiation. These associations remained directionally consistent across supportive and sensitivity analyses, although residual confounding inherent to observational studies cannot be excluded.

## Introduction

Dementia is a leading cause of disability and mortality worldwide, with prevalence expected to rise substantially due to global population aging (1). Despite extensive research, disease-modifying therapies for dementia remain limited, highlighting the importance of identifying pharmacological exposures associated with favorable long-term cognitive outcomes.

Studies indicate that patients with bipolar disorder are increasingly vulnerable to dementia (2–5). The therapeutic management of bipolar disorder and related affective psychoses has long relied on two primary pharmacological pillars: lithium (Li) and valproic acid (VPA). Beyond bipolar disorder, these agents are also widely prescribed for other psychiatric conditions, including schizophrenia and schizoaffective disorder, to manage mood instability, impulsivity, and aggressive behavior. Research also suggests that patients with schizophrenia have an increased risk of developing dementia (2). Although both medications are effective mood stabilizers, their long-term effects on survival and cognitive outcomes remain an area of ongoing clinical interest. Consequently, understanding the long-term outcomes associated with these commonly prescribed mood stabilizers has become increasingly important in clinical practice.

Lithium (Li), a classic mood stabilizer, has been used in psychiatric practice for decades, primarily for bipolar disorder. Experimental and observational studies have suggested that lithium may influence biological pathways implicated in neurodegeneration, including inhibition of glycogen synthase kinase-3β (GSK-3β), attenuation of neuroinflammation, promotion of neurogenesis, and modulation of amyloid-β and tau pathology (3–5). Several epidemiological studies have also reported a lower incidence of dementia among patients exposed to lithium (6, 7). Concerns regarding lithium-associated nephrotoxicity and endocrine adverse effects, particularly thyroid dysfunction, have historically limited its use, especially in older adults (8–10). Nevertheless, lithium continues to be prescribed with careful monitoring in routine clinical practice, underscoring the importance of evaluating its long-term benefits and risks using real-world data (11).

Valproic acid (VPA) is another widely prescribed mood stabilizer and antiepileptic drug. While valproate has been investigated for neuropsychiatric indications, its effects on long-term cognitive outcomes remain controversial (12). Moreover, valproate is well known to be associated with weight gain, insulin resistance, dyslipidemia, and metabolic syndrome, all of which are recognized risk factors for cognitive decline and dementia (13, 14).

Although lithium and valproate are among the most commonly prescribed mood stabilizers, studies directly comparing their long-term risks of mortality and dementia remain limited. Furthermore, observational comparisons between these treatments may be influenced by differences in psychiatric diagnoses, clinical characteristics, and treatment selection. Using the TriNetX Global Collaborative Network, we conducted a retrospective propensity score–matched cohort study comparing adults initiating lithium or valproate. To reduce potential confounding, we excluded patients with neurological conditions strongly associated with dementia, including epilepsy and traumatic brain injury (TBI), and comprehensively balanced demographic, clinical, psychiatric, and treatment-related characteristics between treatment groups. We examined the associations between initiation of lithium versus valproate and the long-term risks of all-cause mortality and incident dementia.

## Methods

### Study design and data source

This multinational retrospective cohort study was conducted using data from the TriNetX Global Collaborative Network, a federated research platform that provides access to de-identified electronic health records from participating academic medical centers and healthcare organizations. The TriNetX network aggregates routinely collected clinical data, including demographic characteristics, diagnoses coded using the International Classification of Diseases, prescription records, laboratory measurements, and procedures mapped to standardized terminologies. At the time of analysis, the Global Collaborative Network comprised 168 participating healthcare organizations. However, the TriNetX analytics platform does not provide information on the geographic distribution or individual contributions of participating organizations. All analyses were performed within the TriNetX analytics environment. The study period spanned from January 1, 2004, to December 31, 2024. All data accessed through TriNetX are de-identified and comply with the Health Insurance Portability and Accountability Act and other applicable international data-protection regulations. The TriNetX platform provides access only to aggregated, de-identified data and does not permit identification of individual patients.

This study protocol was reviewed and approved by the Institutional Review Board of Taichung Veterans General Hospital (IRB No. CE26016B). Because the study involved secondary analysis of de-identified electronic health record data, the requirement for informed consent was waived. The authors had access only to aggregated data available within the TriNetX platform. Data were accessed on December 22, 2025, and all analyses were performed using data available at that time. The study was designed and reported according to the Strengthening the Reporting of Observational Studies in Epidemiology (STROBE) guidelines.

### Study population

Adults aged 40 years or older who initiated treatment with either lithium or valproate during the study period were eligible for inclusion. A new-user design was applied, requiring patients to have no recorded prescription of the comparator medication before treatment initiation. The index date was defined as the date of the first recorded prescription of lithium or valproate.

Patients were excluded if they had a history of dementia, epilepsy or recurrent seizure disorders, traumatic brain injury, kidney transplantation, chronic kidney disease stage 4 or stage 5, end-stage renal disease, acute kidney injury, or an estimated glomerular filtration rate (eGFR) below 30 mL/min/1.73 m^2^ before or on the index date. These exclusion criteria were selected to reduce confounding from neurological disorders strongly associated with dementia and to minimize baseline differences in advanced kidney disease. Patients with previous exposure to the comparator medication were also excluded.

For the primary intention-to-treat analysis, patients were classified into mutually exclusive lithium and valproate cohorts according to their first recorded prescription of the index medication. No second prescription was required for inclusion in the primary analysis.

### Exposure assessment

Exposure was defined using prescription records captured in the electronic health record and identified using standardized medication vocabularies within the TriNetX platform. The primary analysis followed an intention-to-treat approach based on the first recorded prescription of lithium or valproate. Patients were analyzed according to their initial treatment assignment regardless of subsequent treatment discontinuation or switching. No second prescription was required for inclusion in the primary analysis.

As a supportive analysis, a 30-day prescription-confirmed cohort was additionally evaluated, requiring a second prescription of the index medication within 30 days after treatment initiation to reduce potential exposure misclassification associated with a single recorded prescription. This requirement should not be interpreted as evidence of continuous treatment throughout follow-up.

### Outcomes

The primary outcomes were all-cause mortality and incident dementia. All-cause mortality was identified using death records available within participating healthcare organizations in the TriNetX network. Mortality ascertainment was based on the death information available within the TriNetX platform. Incident dementia was defined as the first occurrence of diagnostic codes corresponding to Alzheimer’s disease, vascular dementia, frontotemporal dementia, or other specified or unspecified dementias recorded after the index date. Patients with any dementia diagnosis before the index date were excluded to ensure ascertainment of incident cases.

Follow-up commenced 1 day after the index date and continued until the occurrence of the outcome of interest, death, loss to follow-up (defined as the date of the last recorded clinical encounter during the study period), or the end of the predefined follow-up window, whichever occurred first. The maximum follow-up duration was 5 years (1,825 days). Detailed outcome definitions and coding algorithms are provided in Supplementary Table S1.

### Covariates

Baseline covariates were identified using information available before or on the index date. These included demographic characteristics (age, sex, race, and ethnicity); lifestyle-related conditions (tobacco use and alcohol-related disorders); medical comorbidities (hypertension, type 2 diabetes mellitus, hyperlipidemia, ischemic heart disease, cerebrovascular disease, heart failure, chronic obstructive pulmonary disease, liver disease, obesity, and malignancy); and baseline renal function assessed by eGFR, where laboratory data were available. Missing laboratory values, including eGFR, were not imputed.

To reduce confounding by indication, additional psychiatric and treatment-related characteristics were incorporated into the propensity score model, including bipolar disorder, schizophrenia, schizoaffective disorder, suicidal ideation, healthcare utilization (office or outpatient visits, emergency department visits, hospital inpatient or observation care, and preventive medicine services), and concomitant psychotropic medications (antidepressants, antipsychotics, antiepileptic drugs, lamotrigine, carbamazepine, and oxcarbazepine). Detailed definitions and coding algorithms are provided in Supplementary Table S1.

### Propensity score matching

Propensity scores were estimated using logistic regression incorporating all prespecified baseline demographic, clinical, psychiatric, healthcare utilization, laboratory, and medication variables. Patients initiating lithium were matched 1:1 with patients initiating valproate using nearest-neighbor matching without replacement and a caliper width of 0.1 of the standard deviation of the logit of the propensity score.

Covariate balance before and after matching was evaluated using standardized mean differences (SMDs), with values below 0.10 considered indicative of adequate balance.

### Statistical analysis

Baseline characteristics were summarized as means with standard deviations for continuous variables and counts with percentages for categorical variables. Time-to-event analyses were performed using Cox proportional hazards models to estimate hazard ratios (HRs) and 95% confidence intervals (CIs) comparing lithium and valproate. Kaplan–Meier methods were used to estimate cumulative event probabilities, with group differences assessed using the log-rank test.

Prespecified subgroup analyses for the primary outcomes were conducted according to age, sex, baseline renal function, and major comorbid conditions within the propensity score–matched cohort without additional propensity score matching. Hazard ratios and 95% confidence intervals were estimated separately within each subgroup using Cox proportional hazards models. Formal interaction tests were performed to evaluate effect modification across prespecified subgroup variables, and the corresponding *p* values for interaction are reported. All statistical tests were two-sided, and a *p* < 0.05 was considered statistically significant.

Covariate balance before and after propensity score matching was evaluated using standardized mean differences (SMDs), with values <0.10 considered indicative of adequate balance. Covariate balance is presented numerically in Table 1 and graphically in a Love plot (Supplementary Figure S1).

**Table 1:** Baseline characteristics of lithium and valproate initiators before and after propensity score matching in the primary intention-to-treat cohort.

Characteristics	Before matching						After matching					
	Lithium		Valproate		*p*	SMD	Lithium		Valproate		*p*	SMD
Total number	22,104		644,586				22,098		22,098			
Age at Index	53.9	±10.2	58.1	±11.5	<0.001	0.391	53.9	±10.2	53.8	±10.3	0.575	0.005
Female, n(%)	12,623	(57.1)	341,795	(53.0)	<0.001	0.082	12,618	(57.1)	12,582	(56.9)	0.729	0.003
Race												
White	13,344	(60.4)	411,292	(63.8)	<0.001	0.071	13,344	(60.4)	13,407	(60.7)	0.540	0.006
Black or African American	1939	(8.8)	116,184	(18.0)	<0.001	0.274	1,939	(8.8)	1,827	(8.3)	0.056	0.018
Asian	352	(1.6)	24,970	(3.9)	<0.001	0.140	352	(1.6)	331	(1.5)	0.418	0.008
Ethnicity												
Not Hispanic or Latino	11,139	(50.4)	297,115	(46.1)	<0.001	0.086	11,136	(50.4)	11,803	(53.4)	<0.001	0.060
Hispanic or Latino	1,276	(5.8)	22,850	(3.5)	<0.001	0.106	1,274	(5.8)	1,251	(5.7)	0.637	0.004
Tobacco use	376	(1.7)	10,255	(1.6)	0.199	0.009	375	(1.7)	342	(1.5)	0.214	0.012
Alcohol related disorders	1,010	(4.6)	19,429	(3.0)	<0.001	0.081	1,010	(4.6)	942	(4.3)	0.115	0.015
Comorbidity												
Hypertensive diseases	3,041	(13.8)	149,060	(23.1)	<0.001	0.243	3,041	(13.8)	2,828	(12.8)	0.003	0.028
Type 2 diabetes mellitus	1,425	(6.4)	67,570	(10.5)	<0.001	0.145	1,425	(6.4)	1,349	(6.1)	0.136	0.014
Hyperlipidemia	2,239	(10.1)	100,582	(15.6)	<0.001	0.164	2,237	(10.1)	2,098	(9.5)	0.026	0.021
Neoplasms	1,396	(6.3)	82,970	(12.9)	<0.001	0.224	1,396	(6.3)	1,266	(5.7)	0.009	0.025
Ischemic heart diseases	659	(3.0)	36,335	(5.6)	<0.001	0.131	659	(3.0)	629	(2.8)	0.396	0.008
Cerebrovascular diseases	365	(1.7)	33,261	(5.2)	<0.001	0.194	365	(1.7)	325	(1.5)	0.125	0.015
Heart failure	188	(0.9)	16,747	(2.6)	<0.001	0.135	188	(0.9)	176	(0.8)	0.528	0.006
Obesity	1,208	(5.5)	45,821	(7.1)	<0.001	0.068	1,208	(5.5)	1,139	(5.2)	0.143	0.014
Liver disease	588	(2.7)	22,338	(3.5)	<0.001	0.047	588	(2.7)	555	(2.5)	0.323	0.009
COPD	573	(2.6)	25,389	(3.9)	<0.001	0.076	573	(2.6)	577	(2.6)	0.905	0.001
Bipolar disorder	4,909	(22.2)	40,168	(6.2)	<0.001	0.470	4,903	(22.2)	4,416	(20.0)	<0.001	0.054
Schizophrenia	767	(3.5)	15,842	(2.5)	<0.001	0.060	767	(3.5)	787	(3.6)	0.605	0.005
Schizoaffective disorder	691	(3.1)	12,894	(2.0)	<0.001	0.071	691	(3.1)	698	(3.2)	0.849	0.002
Suicidal ideation	1,295	(5.9)	14,203	(2.2)	<0.001	0.187	1,291	(5.8)	1,181	(5.3)	0.023	0.022
Medical utilization, n (%)												
Office or Other Outpatient Services	2,843	(12.9)	170,411	(26.4)	<0.001	0.347	2,843	(12.9)	2,647	(12.0)	0.005	0.027
Hospital Inpatient and Observation Care Services	1,267	(5.7)	54,487	(8.5)	<0.001	0.106	1,267	(5.7)	1,216	(5.5)	0.292	0.010
Emergency Department Services	3,094	(14.0)	108,195	(16.8)	<0.001	0.077	3,094	(14.0)	3,091	(14.0)	0.967	<0.001
Preventive Medicine Services	292	(1.3)	22,361	(3.5)	<0.001	0.141	292	(1.3)	278	(1.3)	0.555	0.006
Lab data, n(%)												
eGFR, mL/min/1.73 m^2^	90.3	±18.5	86.9	±20.0	<0.001	0.177	90.3	±18.5	91.7	±19.1	<0.001	0.072
<60	647	(2.9)	43,970	(6.8)	<0.001	0.182	647	(2.9)	607	(2.7)	0.252	0.011
60-89	3,630	(16.4)	146,298	(22.7)	<0.001	0.159	3,630	(16.4)	3,223	(14.6)	<0.001	0.051
≥90	4,632	(21.0)	159,403	(24.7)	<0.001	0.090	4,629	(20.9)	4,422	(20.0)	0.015	0.023
Medications, n (%)												
Antidepressants	5,106	(23.1)	126,864	(19.7)	<0.001	0.083	5,101	(23.1)	4,665	(21.1)	<0.001	0.048
Antipsychotics	5,381	(24.3)	98,230	(15.2)	<0.001	0.230	5,375	(24.3)	5,227	(23.7)	0.099	0.016
Antiepileptics	4,045	(18.3)	119,720	(18.6)	0.304	0.007	4,039	(18.3)	3,695	(16.7)	<0.001	0.041
Lamotrigine	984	(4.5)	7,554	(1.2)	<0.001	0.199	978	(4.4)	824	(3.7)	<0.001	0.035
Carbamazepine	180	(0.8)	9,222	(1.4)	<0.001	0.059	180	(0.8)	166	(0.8)	0.450	0.007
Oxcarbazepine	218	(1.0)	3,342	(0.5)	<0.001	0.054	216	(1.0)	199	(0.9)	0.402	0.008

Abbreviations: COPD: Chronic Obstructive Pulmonary Disease; eGFR, estimated glomerular filtration rate; PSM, propensity score matching; SMD, standardized mean difference.

All analyses were performed using the built-in analytics tools available within the TriNetX platform. Cohort definitions, inclusion and exclusion criteria, covariates, and outcome definitions were prespecified using standardized clinical coding systems. Detailed coding algorithms are provided in Supplementary Table S1.

### Supportive and sensitivity analyses

Two additional sets of analyses were conducted to evaluate the consistency of the primary findings. First, a supportive 30-day prescription-confirmed analysis was performed by requiring a second prescription of the index medication within 30 days after treatment initiation. This analysis was intended to reduce potential exposure misclassification associated with a single recorded prescription.

Second, lag-time sensitivity analyses using 6-, 12-, and 24-month lag periods were performed to evaluate the potential influence of early events and reverse causation. For each lag analysis, follow-up and outcome ascertainment began after the corresponding lag period among patients remaining eligible in the propensity score–matched cohort. Hazard ratios and 95% confidence intervals were estimated using Cox proportional hazards models.

Results of the supportive prescription-confirmed and lag-time analyses are presented in Supplementary Figures S2, S3, respectively.

### Role of the funding source

The funding sources had no role in the study design, data collection, data analysis, data interpretation, writing of the report, or the decision to submit the manuscript for publication. The corresponding authors had full access to the aggregated data available within the TriNetX platform and had final responsibility for the decision to submit the manuscript for publication.

## Results

### Study population and cohort assembly

Figure 1 illustrates the cohort selection process and propensity score matching for the primary intention-to-treat analysis. From the TriNetX Global Collaborative Network, adults aged ≥40 years initiating lithium or valproate between 2004 and 2024 were identified. After applying the predefined exclusion criteria, patients with pre-existing dementia, epilepsy, traumatic brain injury, advanced chronic kidney disease, acute kidney injury, kidney transplantation, or previous exposure to the comparator medication were excluded. The primary intention-to-treat cohort included 22,098 patients in each treatment group after 1:1 propensity score matching.

**Figure 1:**
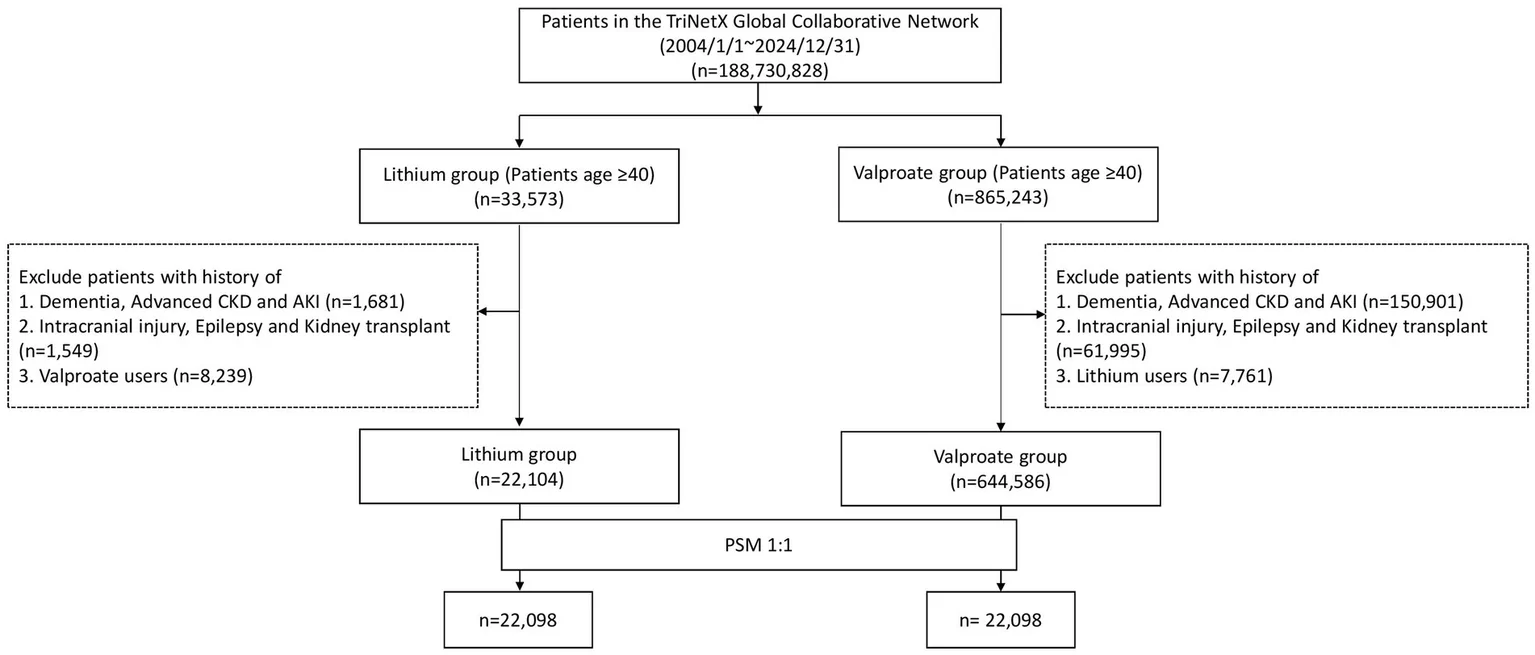
Cohort selection and construction of the propensity score–matched cohort for the primary intention-to-treat analysis. Flow diagram illustrating patient identification from the TriNetX Global Collaborative Network, application of the predefined inclusion and exclusion criteria, and construction of the propensity score–matched cohorts. Adults aged ≥40 years with a first recorded prescription of lithium or valproate were classified according to their initial treatment. Patients with pre-existing dementia, epilepsy, intracranial injury, kidney transplantation, advanced chronic kidney disease, acute kidney injury, or previous exposure to the comparator medication were excluded. No second prescription was required for inclusion in the primary intention-to-treat analysis. Propensity score matching was subsequently performed in a 1:1 ratio, resulting in 22,098 patients in each treatment group.

### Baseline characteristics

Baseline characteristics before and after propensity score matching are summarized in Table 1. Before matching, lithium initiators were generally younger and had a lower prevalence of cardiometabolic comorbidities than valproate initiators. After propensity score matching, demographic characteristics, medical comorbidities, psychiatric diagnoses, healthcare utilization, laboratory measurements, and concomitant medications were well balanced between the two cohorts, with all standardized mean differences below 0.10.

### Primary outcomes: all-cause mortality and incident dementia

Figure 2 presents the primary outcome analyses in the propensity score–matched intention-to-treat cohort. During follow-up, 884 deaths occurred among lithium initiators and 1,585 among valproate initiators. The corresponding five-year Kaplan–Meier-estimated cumulative event probabilities for all-cause mortality were 6.61 and 10.65%, respectively. Lithium initiation was associated with a lower recorded risk of all-cause mortality than valproate initiation (HR 0.58, 95% CI 0.53–0.63; *p* < 0.001).

**Figure 2:**
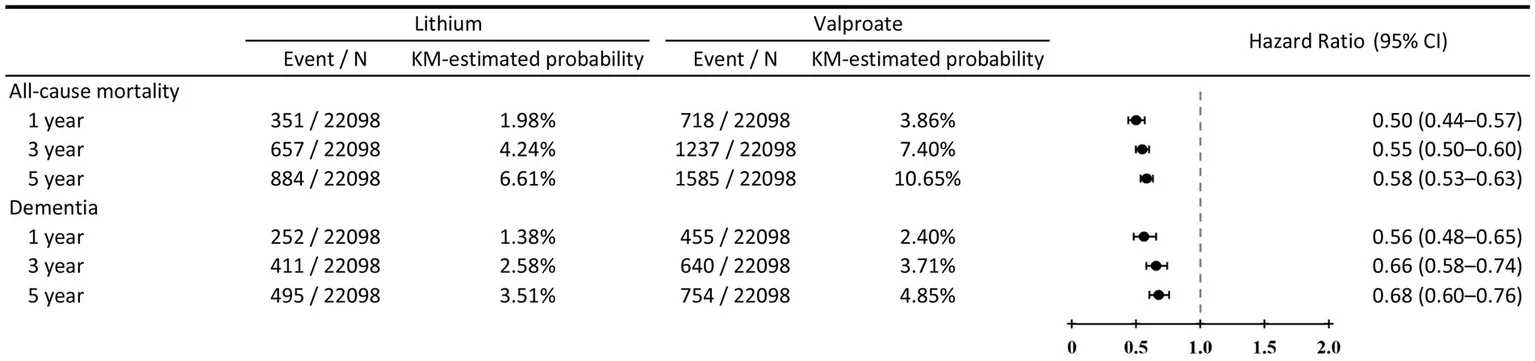
Primary intention-to-treat analysis of all-cause mortality and incident dementia following lithium versus valproate initiation. Forest plot showing hazard ratios (*HRs*), 95% confidence intervals (*CIs*), event counts, and Kaplan–Meier-estimated cumulative event probabilities for all-cause mortality and incident dementia at 1, 3, and 5 years in the propensity score–matched intention-to-treat cohort. Patients were classified according to their first recorded prescription of lithium or valproate without requiring a second prescription after treatment initiation. *HRs* were estimated using Cox proportional hazards models. Hazard ratios below 1 indicate lower recorded risks associated with lithium initiation.

Incident dementia occurred in 495 lithium initiators and 754 valproate initiators. The corresponding 5-year Kaplan–Meier-estimated cumulative event probabilities for incident dementia were 3.51 and 4.85%, respectively. Lithium initiation was also associated with a lower recorded risk of incident dementia (HR 0.68, 95% CI 0.60–0.76; *p* < 0.001).

Kaplan–Meier-estimated cumulative event probability curves for all-cause mortality and incident dementia are shown in Figure 3. Separation between the lithium and valproate groups became apparent early after treatment initiation and persisted throughout follow-up, with lower cumulative event probabilities among lithium initiators for both outcomes (log-rank *p* < 0.001).

**Figure 3:**
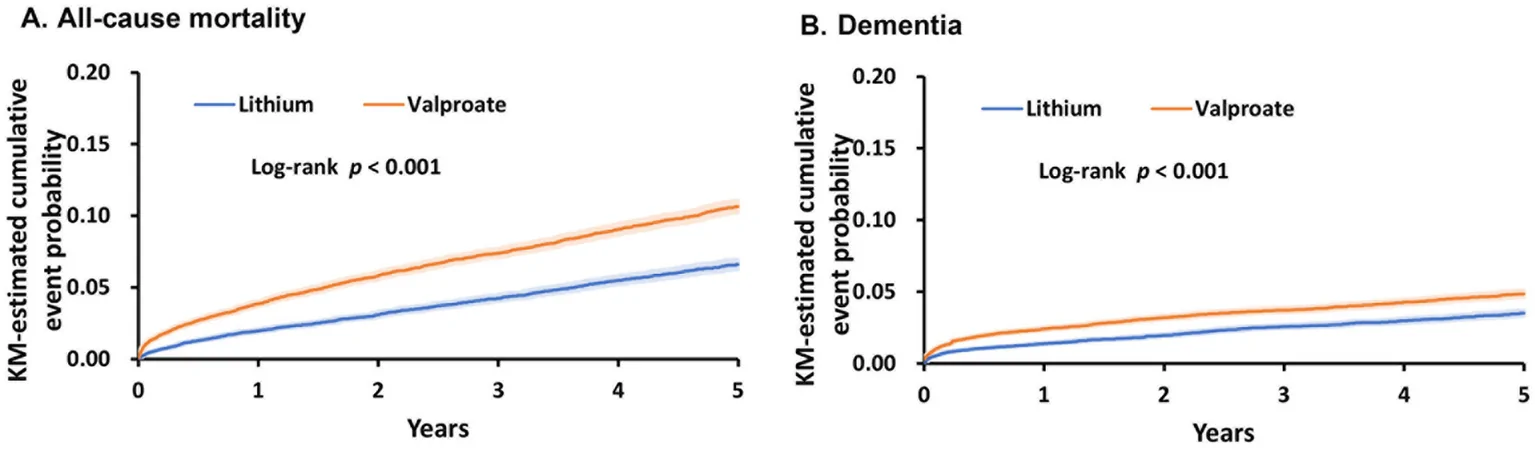
Kaplan–Meier-estimated cumulative event probability curves for all-cause mortality and incident dementia. Kaplan–Meier-estimated cumulative event probability curves for **(A)** all-cause mortality and **(B)** incident dementia among patients initiating lithium or valproate in the propensity score–matched intention-to-treat cohort. Patients were classified according to their first recorded prescription. Group differences were assessed using the log-rank test. Shaded areas represent 95% confidence intervals. Time zero corresponds to the index date.

### Subgroup analyses for all-cause mortality

Figure 4 presents subgroup analyses for all-cause mortality in the propensity score–matched intention-to-treat cohort. The association between lithium initiation and lower recorded all-cause mortality was directionally consistent across prespecified subgroups. Statistically significant interactions were observed for age, race, and hypertension status (P for interaction = 0.016, 0.011, and 0.012, respectively), whereas no statistically significant interactions were observed for sex, type 2 diabetes mellitus, hyperlipidemia, or obesity.

**Figure 4:**
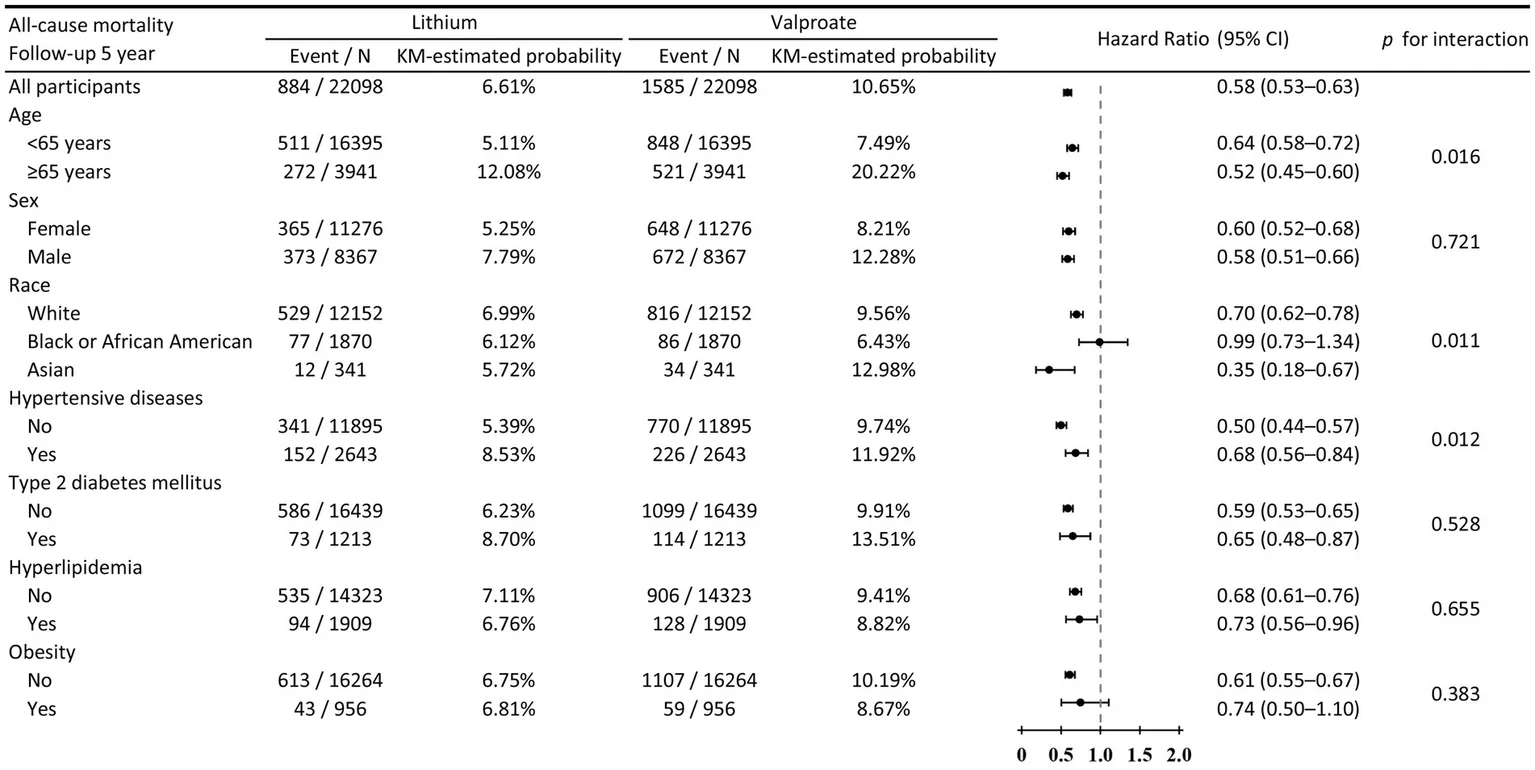
Subgroup analyses of the association between lithium versus valproate initiation and all-cause mortality. Forest plot showing hazard ratios (*HRs*) and 95% confidence intervals (*CIs*) for all-cause mortality across prespecified subgroups in the propensity score–matched intention-to-treat cohort. Hazard ratios were estimated separately within each subgroup using Cox proportional hazards models without additional propensity score matching. Formal interaction tests were performed to evaluate effect modification across subgroup variables, and the corresponding *p*-values for interaction are presented.

### Subgroup analyses for incident dementia

Subgroup analyses for incident dementia are presented in Figure 5. The association between lithium initiation and lower recorded incident dementia was directionally consistent across prespecified subgroups. A statistically significant interaction was observed for hypertension status (P for interaction = 0.002), whereas no statistically significant interactions were observed for age, sex, race, type 2 diabetes mellitus, hyperlipidemia, or obesity.

**Figure 5:**
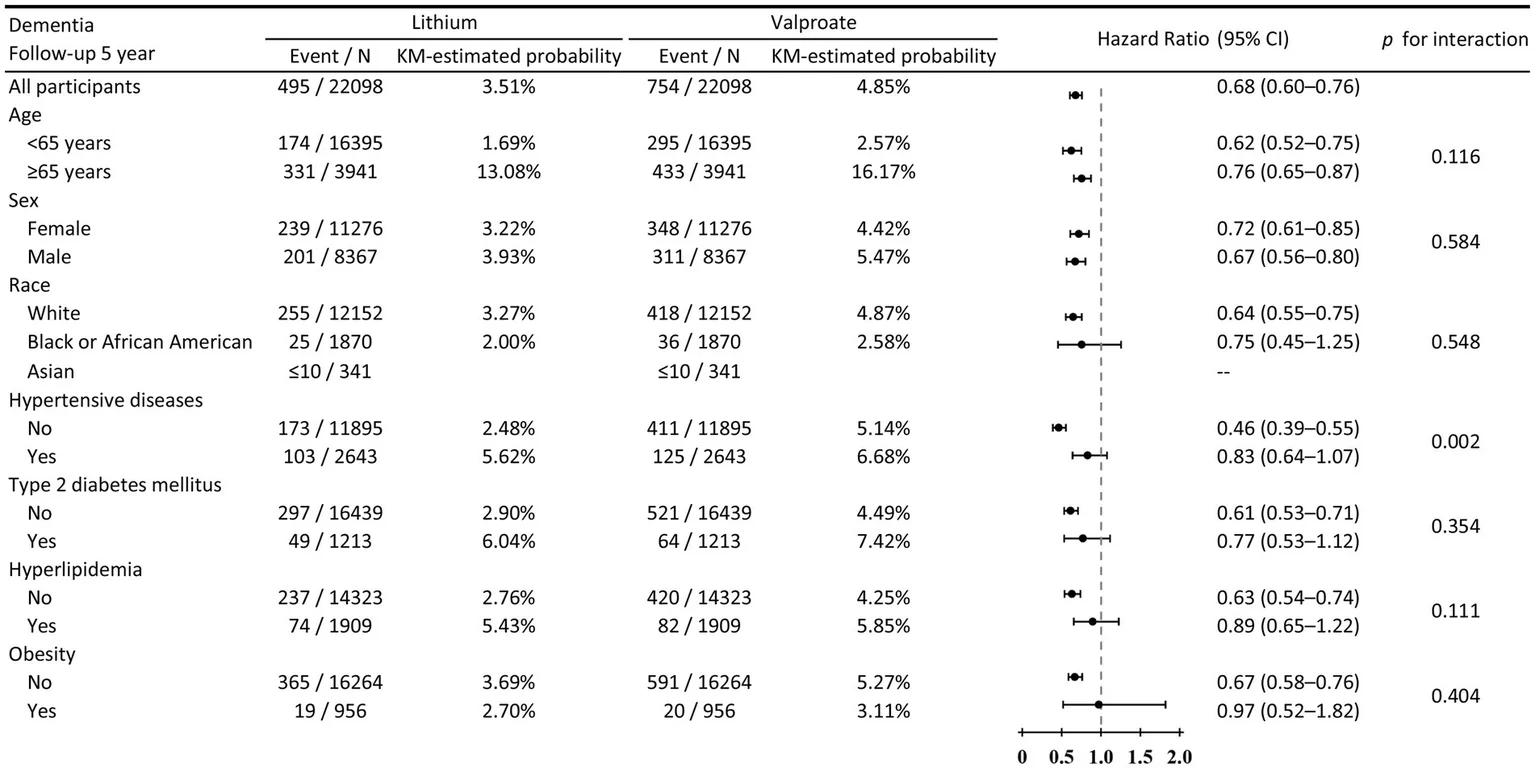
Subgroup analyses of the association between lithium versus valproate initiation and incident dementia. Forest plot showing hazard ratios (HRs) and 95% confidence intervals (CIs) for incident dementia across prespecified subgroups in the propensity score–matched intention-to-treat cohort. Hazard ratios were estimated separately within each subgroup using Cox proportional hazards models without additional propensity score matching. Formal interaction tests were performed to evaluate effect modification across subgroup variables, and the corresponding *p-*values for interaction are presented.

### Supportive and sensitivity analyses

Results of the supportive 30-day prescription-confirmed analysis and lag-time sensitivity analyses are presented in Supplementary Figures S2, S3, respectively. In the supportive prescription-confirmed analysis, the direction and magnitude of the associations with all-cause mortality and incident dementia were consistent with those observed in the primary intention-to-treat analysis. The 6-, 12-, and 24-month lag-time analyses similarly yielded directionally consistent findings.

## Discussion

In this multinational real-world cohort study using the TriNetX Global Collaborative Network, lithium initiation was associated with lower recorded risks of incident dementia and all-cause mortality compared with valproate among adults aged 40 years or older after excluding patients with epilepsy and traumatic brain injury, as well as other major baseline neurological and renal conditions. These findings suggest that lithium initiation is associated with more favorable recorded long-term survival and dementia outcomes than valproate initiation in routine clinical practice, although causal effects cannot be inferred (1–5).

The observed lower dementia risk among lithium users is consistent with prior population-based studies suggesting an association between lithium exposure and reduced dementia incidence (6, 15, 16). The biological plausibility of lithium’s neuroprotective association is supported by experimental and translational literature implicating inhibition of glycogen synthase kinase-3β (GSK-3β), a kinase involved in tau hyperphosphorylation and amyloid-β accumulation in Alzheimer’s disease (3–5, 17, 18). Lithium has also been reported to enhance neurotrophic and anti-apoptotic signaling, including upregulation of BDNF and Bcl-2, and to influence neurogenesis and structural brain correlates relevant to cognition (19–21). In contrast, valproate has a well-established propensity to induce weight gain and metabolic disturbances, including insulin resistance and dyslipidemia (13, 14, 22, 23). Because metabolic syndrome and midlife cardiometabolic risk factors are strongly associated with later-life cognitive decline and dementia, these pathways may partially contribute to the higher dementia incidence observed in the valproate cohort (24–26).

A key design feature of this study was the exclusion of epilepsy and traumatic brain injury, which strengthens interpretability of the dementia findings by reducing confounding from neurological conditions that are independently associated with increased dementia risk and may also influence treatment choice (27–29). Valproate is commonly used in patients with epilepsy and post-traumatic seizure risk, populations that may be particularly vulnerable to cognitive decline, and inclusion of these patients could bias comparative estimates if not appropriately addressed (27–29). By restricting to patients without these conditions at baseline, the present analysis improves cohort comparability and reduces the likelihood that the observed association is driven primarily by underlying neurological vulnerability rather than differential treatment-related risk (27–29).

The lower mortality risk observed with lithium compared with valproate aligns with prior evidence indicating lithium’s superiority in reducing suicide-related outcomes and potentially all-cause mortality in mood disorders (30, 31). Lithium’s anti-suicidal effect has been consistently supported across randomized trials, meta-analyses, and large observational datasets, whereas evidence for valproate in suicide prevention is less consistent (30, 31). Although the mechanisms underlying lithium’s anti-suicidal properties remain incompletely defined, inflammatory pathways may be relevant; elevated C-reactive protein and pro-inflammatory cytokines have been associated with suicidal behavior in mood disorders, and lithium has been linked to modulation of inflammatory and oxidative stress signaling, including via GSK-3-related pathways (32–36). Beyond suicide prevention, lithium has been associated with lower risks of certain chronic conditions that contribute to premature mortality, including selected cancers and osteoporosis, and experimental data suggest possible vascular benefits through pathways related to elastin biology and aneurysm suppression, although these observations should be interpreted cautiously (37–39). Finally, lithium remains among the most effective agents for long-term relapse prevention in classic bipolar disorder, and better relapse control may reduce cumulative exposure to periods of heightened behavioral and medical risk, thereby contributing to improved survival (40). The relatively high five-year mortality probabilities observed in both treatment groups (6.61% in the lithium group and 10.65% in the valproate group), as estimated using the Kaplan–Meier method, should be interpreted in the context of the underlying clinical characteristics of this population. Patients requiring mood-stabilizing treatment may carry substantial psychiatric and medical morbidity that is not fully captured by structured electronic health record variables. In addition, treatment selection may reflect unmeasured differences in illness severity, frailty, or clinician prescribing patterns despite propensity score matching. Mortality ascertainment in a federated electronic health record network also depends on death information available to participating healthcare organizations and may therefore be incomplete or heterogeneous across healthcare systems. These factors may have contributed to both the absolute mortality rates and the observed between-group difference, and the mortality findings should therefore be interpreted cautiously.

This study has several strengths. It leveraged a large multinational electronic health record network with extended follow-up and applied a new-user design with propensity score matching to improve comparability between treatment groups and reduce confounding by indication. In addition, the exclusion of epilepsy and traumatic brain injury strengthened the dementia analysis by minimizing confounding from neurological conditions strongly associated with cognitive outcomes and treatment selection (27–29). The large sample size and generally consistent direction of associations across subgroup analyses further support the overall findings, although evidence of effect modification was observed for selected subgroups. These subgroup findings should be considered exploratory given the multiple subgroup comparisons and should not be interpreted as evidence of differential causal treatment effects.

Nevertheless, several limitations should be considered. First, as an observational study based on electronic health records, residual confounding cannot be completely excluded despite the use of propensity score matching. Important clinical variables such as illness severity, psychiatric subtype, mood episode polarity, and clinician prescribing preferences were not fully captured in the database and may have influenced treatment selection. Second, medication exposure was inferred from prescription records and may not fully reflect actual medication adherence. The primary analysis followed an intention-to-treat approach based on the first recorded prescription; therefore, subsequent treatment discontinuation, switching, or changes in adherence were not captured in the exposure definition. A supportive 30-day prescription-confirmed analysis yielded directionally consistent findings, although requiring a second prescription may itself introduce selection related to early treatment continuation. Information on lithium dose, serum lithium concentrations, treatment duration, and cumulative exposure was not available, precluding assessment of dose–response relationships. Third, diagnostic misclassification is possible because outcomes were identified using diagnostic codes recorded in routine clinical practice. Differences in healthcare utilization and monitoring intensity between treatment groups may also influence outcome detection. Because follow-up was based on routinely collected electronic health records, patients without subsequent clinical encounters were considered lost to follow-up at the date of their last recorded healthcare encounter. In addition, the TriNetX built-in analytics environment did not support cumulative incidence functions or competing-risk regression, and individual-level time-to-event data were not available for external analysis. Dementia estimates were therefore derived using conventional Cox proportional hazards models and should be interpreted cautiously in the presence of the competing risk of death. Fourth, although this study used a large multinational electronic health record network, the geographic composition and relative contribution of participating healthcare organizations are not available within the TriNetX platform. Consequently, the generalizability of these findings to individual healthcare systems should be interpreted with caution. Finally, because this study relied on observational real-world data, causal relationships cannot be established. The findings should therefore be interpreted as comparative effectiveness associations rather than causal effects. Future studies incorporating more detailed clinical information, medication exposure data, competing-risk analyses, and prospective study designs are warranted to further clarify the potential mechanisms underlying these associations.

In conclusion, in this multinational real-world cohort study, lithium initiation was associated with lower recorded risks of incident dementia and all-cause mortality compared with valproate initiation among adults aged 40 years or older without baseline epilepsy or traumatic brain injury. These findings provide real-world evidence suggesting differential long-term outcomes associated with lithium and valproate treatment in routine clinical practice. Further research incorporating richer clinical data and prospective designs is needed to better understand the mechanisms underlying these associations.

## Data Availability

The datasets presented in this article are not readily available because the data used in this study were obtained from the TriNetX Global Collaborative Network and are subject to data use agreements and institutional restrictions. Requests to access the datasets should be directed to TriNetX Global Collaborative Network, https://trinetx.com, contact@trinetx.com.
