# Supplementary material for: Association of lithium versus valproate initiation with all-cause mortality and incident dementia: a multinational real-world cohort study
Source: Front Neurol. 2026 Sep 22;17:1847655. doi: 10.3389/fneur.2026.1847655 (PMC13638382; doi:10.3389/fneur.2026.1847655)
Supplement: Supplementary file 1 [file Supplementary_file_1.docx]

**Supplementary Material**

**Association of lithium versus valproate initiation with all-cause mortality and incident dementia: a multinational real-world cohort study**

Da-Han Hou, Yu-Hsien Wu, Ming-Ju Wu, Shiau-Shian Huang, Po-Hsun Hou

**Contents**

Supplementary Table S1. ICD-10, CPT, LOINC, ATC, and RxNorm codes used in the study
Supplementary Figure S1. Covariate balance before and after propensity score matching in the primary intention-to-treat analysis (Love plot)
Supplementary Figure S2. Supportive 30-day prescription-confirmed analysis
Supplementary Figure S3. Lag-time sensitivity analyses
Supplementary Figure S4. Risk of incident hypothyroidism and hypercalcemia

**Supplementary Table S1. ICD-10, CPT, LOINC, ATC, and RxNorm codes used in the study**

Definitions and standardized clinical coding systems used for treatment exposure, baseline exclusion criteria, propensity score matching covariates, healthcare utilization variables, laboratory measurements, concomitant medications, and study outcomes. ICD-10-CM, CPT, LOINC, ATC, and RxNorm coding systems were used as appropriate.

| **Treatment exposure** | |
| --- | --- |
| Lithium | ATC N05AN |
| Valproate | RXNORM:40254 |
| **Baseline exclusion criteria** | |
| Vascular dementia | ICD-10-CM F01 |
| Dementia in other diseases classified elsewhere | ICD-10-CM F02 |
| Unspecified dementia | ICD-10-CM F03 |
| Alcohol dependence with alcohol-induced persisting dementia | ICD-10-CM F10.27 |
| Alzheimer's disease | ICD-10-CM G30 |
| Frontotemporal dementia | ICD-10-CM G31.0 |
| Neurocognitive disorder with Lewy bodies | ICD-10-CM G31.83 |
| Kidney transplant status | ICD-10-CM Z94.0 |
| Chronic kidney disease, stage 4 | ICD-10-CM N18.4 |
| Chronic kidney disease, stage 5 | ICD-10-CM N18.5 |
| End stage renal disease | ICD-10-CM N18.6 |
| eGFR<30 | LOINC 98979-8 |
| Acute kidney failure | ICD-10-CM N17 |
| Intracranial injury | ICD-10-CM S06 |
| Epilepsy and recurrent seizures | ICD-10-CM G40 |
| **Propensity score matching covariates** | |
| Hypertensive diseases | ICD-10-CM I10-I1A |
| Type 2 diabetes mellitus | ICD-10-CM E11 |
| Hyperlipidemia | ICD-10-CM E78 |
| Neoplasms | ICD-10-CM C00-D49 |
| Ischemic heart diseases | ICD-10-CM I20-I25 |
| Cerebrovascular diseases | ICD-10-CM I60-I69 |
| Heart failure | ICD-10-CM I50 |
| Obesity | ICD-10-CM E66 |
| Liver disease | ICD-10-CM K70-K77 |
| Chronic obstructive pulmonary disease | ICD-10-CM J44 |
| Bipolar disorder | ICD-10-CM F31 |
| Schizophrenia | ICD-10-CM F20 |
| Schizoaffective disorder | ICD-10-CM F25 |
| Suicidal ideation | ICD-10-CM R45.851 |
| Office or other outpatient services | CPT 1013626 |
| Hospital Inpatient and Observation Care Services | CPT 1013829 |
| Emergency Department Services | CPT 1013711 |
| Preventive Medicine Services | CPT 1013659 |
| Tobacco use | ICD-10-CM Z72.0 |
| Alcohol related disorders | ICD-10-CM F10 |
| eGFR | LOINC 98979-8 |
| Antidepressants | ATC N06A |
| Antipsychotics | ATC N05A |
| Antiepileptics | ATC N03A |
| Lamotrigine | RxNorm 28439 |
| Carbamazepine | RxNorm 2002 |
| Oxcarbazepine | RxNorm 32624 |
| **Outcome** | |
| All-cause mortality | Ill-defined and unknown cause of mortality (ICD-10-CM R99) Deceased |
| Dementia | Vascular dementia (ICD-10-CM F01)  Dementia in other diseases classified elsewhere (ICD-10-CM F02)  Unspecified dementia (ICD-10-CM F03)  Alcohol dependence with alcohol-induced persisting dementia (ICD-10-CM F10.27)  Alzheimer's disease (ICD-10-CM G30)  Frontotemporal dementia (ICD-10-CM G31.0) |

**Supplementary Figure S1. Covariate balance before and after propensity score matching in the primary intention-to-treat analysis (Love plot).**

Absolute biting standardized mean differences (SMDs) for baseline covariates included in the propensity score model before and after propensity score matching in the primary intention-to-treat analysis. Covariates included demographic characteristics, medical comorbidities, psychiatric diagnoses, healthcare utilization, laboratory measurements, and concomitant medications. The vertical dashed line indicates the conventional threshold of an absolute SMD of 0.10 for acceptable covariate balance. All measured covariates had post-matching absolute SMDs below 0.10, indicating adequate balance between the lithium and valproate cohorts.

**Supplementary Figure S2. Supportive 30-day prescription-confirmed analysis of the association between lithium and valproate initiation and the risks of all-cause mortality and incident dementia.**

Forest plot showing hazard ratios (HRs) and 95% confidence intervals (CIs) for all-cause mortality and incident dementia in the supportive 30-day prescription-confirmed analysis. Cohorts included patients with a second recorded prescription of the index medication within 30 days after treatment initiation. HRs were estimated using Cox proportional hazards models after propensity score matching. The associations remained directionally consistent with those observed in the primary intention-to-treat analysis.

**Supplementary Figure S3. Lag-time sensitivity analyses of the association between lithium and valproate initiation and the risks of all-cause mortality and incident dementia**

Forest plot showing hazard ratios (HRs) and 95% confidence intervals (CIs) for all-cause mortality and incident dementia using 6-, 12-, and 24-month lag periods after treatment initiation. For each lag analysis, follow-up and outcome ascertainment began after the corresponding lag period among eligible patients remaining in the propensity score–matched cohort. HRs were estimated using Cox proportional hazards models. These analyses were conducted to evaluate the potential influence of early events and reduce the possibility of reverse causation. The direction and magnitude of the associations remained consistent with those observed in the primary intention-to-treat analysis.

**Supplementary Figure S4. Risk of incident hypothyroidism and hypercalcemia following initiation of lithium versus valproate**

Forest plot showing hazard ratios (HRs) and 95% confidence intervals (CIs) for incident hypothyroidism and hypercalcemia comparing lithium and valproate in the propensity score–matched cohort. Patients with pre-existing hypothyroidism or hypercalcemia before the index date were excluded from the corresponding analyses. Cumulative incidences at 1, 3, and 5 years are presented for each outcome, and HRs were estimated using Cox proportional hazards models. Hazard ratios greater than 1 indicate higher recorded risks associated with lithium initiation.
